# College Commencement Exercises

**Published:** 1887-07

**Authors:** 


### EDUCATIONAL.

### Baltimore College of Dental Surgery.

The forty-seventh annual commencement of the Baltimore College of Dental Surgery was held at the Academy of Music, Baltimore, Md., on Thursday, March 10th, 1887, at 1.30 p. m.

The degree of D.D.S. was conferred upon the following graduates by Prof. R. B. Winder, Dean of the Faculty:

| NAME. | RESIDENCE. | NAME. | RESIDENCE. |
|---|---|---|---|
| A. F. Ambrose | Pennsylvania. | A. F. Lantz | New York. |
| S. Ashbrook | Pennsylvania. | G. W. McDonald | Nova Scotia. |
| A. E. Barolet | Connecticut. | M. M. Mixon | Georgia. |
| J. H. Bitzer | Virginia. | E. W. Moyer | Pennsylvania. |
| B. Brodnax | Tennessee. | A. L. Munroe | Florida. |
| S. L. Butler | Virginia. | W. A. Neece | Illinois. |
| G. W. Conner | Kentucky. | F. R. Parramore | Georgia. |
| E. H. Coolbaugh | Pennsylvania. | F. M. Poffenberger | Pennsylvania. |
| J. P. Canfield | Maryland. | J. B. Powers | New Hampshire. |
| T. W. Cowan | Canada. | S. Pugsley | New Brunswick. |
| E. E. Cruzen | Maryland. | J. Roach | Georgia. |
| B. W. Cubbedge | Georgia. | R. Roach | Georgia. |
| D. H. Day | Minnesota. | J. J. Sarrazin | Louisiana. |
| J. S. B. Egan | New York. | C. F. Shine | Florida. |
| H. Floris | Germany. | I. Standiford | Pennsylvania. |
| G. E. Furbay | Ohio. | J. B. L. Swentzel | New York. |
| J. E. Getty | Maryland. | W. S. Twilley | Maryland. |
| B. H. Hawley | Maine. | G. A. Urling | Pennsylvania. |
| H. B. F. Heath | Dist. of Columbia. | H. Urling | Pennsylvania. |
| H. Heime | Massachusetts. | S. B. Ward | Virginia. |
| J. N. Hester | North Carolina. | G. L. Webb | New York. |
| D. M. Hitch | Delaware. | J. Wise | Pennsylvania. |
| F. M. Kennedy | Massachusetts. | J. E. Wyche | North Carolina. |
| H. Koenaart | Belgium. | | |

### Kansas City Dental College.

The annual commencement exercises of the Kansas City Dental College were held in Masonic Hall, Kansas City, Mo., on Tuesday evening, March 15, 1887.

The degree of D.D.S. was conferred on the following graduates:

| NAME. | RESIDENCE. | NAME. | RESIDENCE. |
|---|---|---|---|
| G. W. Earl | Washington Ter. | W. A. McCarter | Indiana. |
| James M. Gross | Missouri. | S. S. Noble | Pennsylvania. |
| J. W. Heckler | Ohio. | J. H. Parsons | Indiana. |
| Robert Lawrence | Indiana. | Frank Strickland | Ohio. |

The honorary degree of D.D.S. was conferred on Dr. H. W. Howe, of Lawrence, Kansas.

### Ohio College of Dental Surgery.

The forty-first annual commencement of the Ohio College of Dental Surgery was held in College Hall, Cincinnati, Ohio, on Wednesday evening, March 2, 1887.

The degree of D.D.S. was conferred on the following graduates by G. W. Keely, D.D.S., President of the Board of Trustees:

| NAME. | RESIDENCE. | NAME. | RESIDENCE. |
|---|---|---|---|
| A. W. Black | Indiana. | C. H. Martin | Ohio. |
| L. A. Brown | Minnesota. | E. S. Mathews | England. |
| L. E. Custer. | Ohio. | H. C. Matlack. | Kentucky. |
| L. E. Day | Ohio. | E. J. McCartney | Pennsylvania. |
| G. H. Doulton. | California. | B. A. McGee | Indiana. |
| W. F. Edmonds. | Kentucky. | C. E. Miles | Ohio. |
| J. S. Emery | Ohio. | A. H. Millman | Ohio. |
| J. W. Fisher | Kentucky. | B. A. Mosbey | Indiana. |
| C. H. Green, Jr | Indiana. | W. W. Reed | Ohio. |
| E. S. Griffis | Ohio. | F. L. Rice. | Ohio. |
| M. A. Hadcock | Canada. | J. M. Rutherford | Ohio. |
| B. C. Hinkley | Ohio. | E. J. Schwartz | Ohio. |
| T. L. Johnson. | Ohio. | James Silcott | Ohio. |
| E. S. Keefer | Ohio. | J. J. Werner | Switzerland. |
| Miss M. L. Leininger | Ohio. | | |

The honorary degree of D.D.S. was conferred on the following: H. H. Harrison, J. Rollo Knapp, William Knight, M. D., and Wilhelm Herbst.

### University of Tennessee—Dental Department.

The ninth annual commencement of the Dental Department of the University of Tennessee was held at the Masonic Theatre, Nashville, Tenn., on Tuesday evening, February 22, 1888.

The degree of D.D.S. was conferred on the following graduates by O. P. Temple, A.M., President of the University:

| NAME. | RESIDENCE. | NAME. | RESIDENCE. |
|---|---|---|---|
| B. F. Atwood | Kentucky. | M. J. Masangill | Louisiana. |
| C. L. Averett | Georgia. | Logan J. McLean | Georgia. |
| James W. Bablow | Kentucky. | A. M. Platt | Michigan. |
| W. Fred. Brotherton | Wisconsin. | J. Q. Ramsay | Missouri. |
| Frank A. Brown | Indiana. | E. E. Rust | Wisconsin. |
| James K. Bryant | Kentucky. | H. P. Smith | Kansas. |
| F. M. Fewell | Indiana. | John E. Suber | Mississippi. |
| A. A. Francis | Ohio. | John E. Thomas | Indiana. |
| Marshall Grigg | Tennessee. | W. B. White | Kentucky. |
| E. J. Harris | Indiana. | H. M. Wilcox | Missouri. |
| W. W. Krape | Illinois. | | |

The honorary degree of D.D.S was conferred on N. H. Wilson, of Indiana.

### New York College of Dentistry.

The twenty-first annual commencement of the New York College of Dentistry was held at Chickering Hall, New York City, on Wednesday evening, March 9, 1887.

The degree of D.D.S. was conferred on the following graduates by M. McN. Walsh, Esq., President of the Board of Trustees:

James Charles Alker.

George Sumner Burt.

Gregorio Santos Benety Llata, M.D.

Samuel Skinner Brown.

Valentine Edw. Norman Cook.

Thomas Alfred Clawson.

John Harvey Crane.

John Richard Crawford.

John Clayton Downs.

Frank Perry Denny.

George Anthony Dow.

Frank John Eversfield.

William Eybel.

Samuel Hassell, Jr.

Erastus Otis Houghton.

Spencer Cone Hamilton.

Paul William Hiller.

Halstead Pell Hodson.

Ira Daniel Horton.

Samuel Porter Hopkins.

Leo Frederic Hugle.

Frank Alfred Katzmeier.

Samuel James Kennedy.

Louis Charles Leroy.

Frank Butler Longenecker.

Edwin Parker Marshall.

Francis Joseph McLaren.

Ferdinand Moith.

Henry Middleton.

Lorenzo Noa.

George Edward Nearing.

Arthur German Rouse.

Franklin Willard Rogers.

Dudley John Russell.

Horace Reynolds.

Felix Edmond San Fuentes, Ph. B.

Thomas Howard Stevens.

Charles Harvey Smith.

Harold Slade.

Preston McCready Sharp.

Richard James Secor.

Walter Lincoln Scofield.

Joseph Daniel Sayre.

George Joseph Taylor.

Daniel Webster Valentine.

Walter Woolsey.

Herman Eugen Albert Wichert.

George Mortimer Whitfield.

John Van Pelt Wicks.

Ulysses Grant Woolley.

Leonhardt Eichbery Zuchtmann.

### University of Iowa—Dental Department.

The fifth annual commencement of the Dental Department of the State University of Iowa was held in the Opera House, Iowa City, Iowa, on Monday evening, February 28, 1887.

The degree of D.D.S. was conferred on the following graduates by J. L. Packard, L.L.D., President of the University:

| NAME. | RESIDENCE. |
|---|---|
| C. W. Aydelotte | Indiana. |
| H. N. Edwards | Iowa. |
| L. S. Field | Iowa. |
| E. T. Giddings | Iowa. |
| E. S. Glasier | Iowa. |
| T. J. Glenn | Wisconsin. |
| C. H. Hare | Iowa. |
| F. A. Hefner | Iowa. |
| J. H. Johnson | Iowa. |
| J. J. Little | Iowa. |
| W. A. Maxwell | Iowa. |
| R. McNutt | Iowa. |
| J. A. Neill | Iowa. |
| Jessie Ritchey | Iowa. |
| F. M. Shriver | Iowa. |
| H. W Shriver | Iowa. |
| J. W. Soule | Iowa. |
| Joseph Scott | Iowa. |
| W. R. Tipton | Iowa. |
| H. M. Vawter | Iowa. |
| J. B. Vernon | Iowa. |
| D. P. Wetzel | Iowa. |
| Alfred Wood, A. M. | Iowa. |
| Geo. B. Yergey | Iowa. |

### Meharry Medical College—School of Dentistry.

The eleventh commencement of the Meharry Medical Department and School of Dentistry (first commencement) of the Central Tennessee College was held at the Masonic Theatre, Nashville, Tenn., on Monday, February 21, 1887.

The degree of D.D.S. was conferred on the following graduates in Dentistry by Rev. J. Braden, D.D., President of the College:

| NAME. | RESIDENCE. | NAME. | RESIDENCE. |
|---|---|---|---|
| John W. Anderson, M.D | Kansas. | Robert F. Boyd, M. | Tennessee. |
| Henry T. Noel, M.D. | Tennessee. | | |

### Howard University Dental College.

The annual commencement of the Dental College of Howard University was held in connection with that of the Medical Department, at the Congregational Church, Washington, D. C., on March 9, 1887, at 7 30 p. m.

The degree of D.D.S. was conferred on the following graduates of the Dental Class by W. W. Patton, D.D., L.L.D., President of the University:

| NAME. | RESIDENCE. | NAME. | RESIDENCE. |
|---|---|---|---|
| Charles V. Barrington | Illinois. | T. Ellsworth Lee | New York. |
| B. F. Darling, M.D. | Iowa. | Franz M. A. Reinhard | Germany. |
| Jefferson H. Johnston | Ohio. | Murdoch C. Smith | Massachusetts. |

### Minnesota Hospital College—Dental Department.

The fifth annual commencement of the Dental Department of the Minnesota Hospital College was held, in connection with the Medical Department, in the Hennepin-avenue M. E. Church, Minneapolis, Minn., on Friday evening, March 11, 1887.

The degree of D.D.S. was conferred on the following graduates by C. H. Hunter, A.M., M.D., President of the Faculty:

| NAME. | RESIDENCE. | NAME. | RESIDENCE. |
|---|---|---|---|
| James E. Cummings | New York. | Thomas S. Rounce | Wisconsin. |
| H. Ben. Ober | Connecticut. | Clarence Strachauer | Minnesota. |

### University of Maryland—Dental Department.

The fifth annual commencement of the Dental Department of the University of Maryland was held at the Academy of Music, Baltimore, Md., on Wednesday, March 16, 1887.

The degree of D.D.S. was conferred on the following graduates by Hon. S. Teackle Wallis, L.L.D., Provost of the University:

| NAME. | RESIDENCE. | NAME. | RESIDENCE. |
|---|---|---|---|
| Julius Albrecht | Germany. | C. T. Loving | Texas. |
| Joseph M. Baker | Arkansas. | W. M. Meador, M.D. | South Carolina. |
| Alonzo A. Bemis | Massachusetts. | Samuel McColl | Canada. |
| D. B. Blauvelt | New York. | J. A. H, Miller | West Virginia. |
| Garabed Boyajian | Asiatic Turkey. | W. C. Miller | Pennsylvania. |
| Charles J. Brawner | Georgia. | W. N. Murphy | Texas. |
| Henry E. Chase | Massachusetts. | John H. Neill | New York. |
| J. G. Chisholm | Alabama. | A. L. de Oliveira | Brazil. |
| Fred. J. Crowell | New Hampshire. | H. H. Phillips | Pennsylvania. |
| Henry E. Douglass | New York. | P. A. Rambo | Georgia. |
| John T. Eiker | Dist. Columbia. | S. S. Reamer | Virginia. |
| G. McC. Faulkner | Pennsylvania. | W. F. Richards | Illinois. |
| W. C. Y. Ferguson | Canada. | W. A. Robertson | Canada. |
| C. G. Frantz | Pennsylvania. | Cary C. Sapp | North Carolina. |
| Joel N. Furman | New York. | E. H. Shields | Ohio. |
| H. Garbrecht | Germany. | H. R. Shine | Florida. |
| William F. Gray | Virginia. | R. A. Shine, Jr | Florida. |
| L. Lee Harban | Maryland | E. E. P. Sleppy | Pennsylvania. |
| Edwin L. Harris | Massachusetts. | P. P. Starke | Virginia. |
| Anton J. Hecker | Germany. | R. W. Talbott | Dist. Columbia. |
| S. W. Hoopes | Maryland. | M. W. White | South Carolina. |
| H. V. Horton | North Carolina. | Arnold Wietfeldt | Germany. |
| Michael Hourihane | Virginia. | H. T. Wilhelm | Germany. |
| Max Jaenicke | Germany. | John H. Wilson | New York. |
| Paul Jaenicke | Germany. | W. W. Wogan | Pennsylvania. |
| James L. Kean | Virginia. | | |

### Indiana Dental College.

The eighth annual commencement of the Indiana Dental College was held in the lecture-room of the college, in Ætna Block, Indianapolis, March 2, 1887, at 8 o'clock p. m.

The degree of D.D.S. was conferred on the following graduates by W. L. Heiskell, D.D.S., President of the Board of Trustees:

| NAME. | RESIDENCE. | NAME. | RESIDENCE. |
|---|---|---|---|
| Jas. W. Bates | Michigan. | Jno. H. Evans | Indiana. |
| L. G. Bell | Germany. | T. J. Hood | Kentucky. |
| John H. Bird | Michigan. | Milton Lamb | Indiana. |
| S. N. Blackledge | Indiana. | J. J. Lickly | Michigan. |
| Jno. E. Carmon | Illinois. | Geo. Marbach | Germany. |
| P. W. Earhart | Indiana. | S. Oliver | Pennsylvania. |
| W. N. Easton | Michigan. | Charles Woelz | Indiana. |

### Vanderbilt University—Department of Dentistry.

The eighth annual commencement of the department of Dentistry of Vanderbilt University was held in the University Chapel, Nashville, Tenn., February 24, 1887.

The degree of D.D.S. was conferred on the following graduates by Chancellor Garland:

| NAME. | RESIDENCE. |
|---|---|
| F. M. Alexander | Georgia. |
| J. J. Austermell | Missouri. |
| T. B. Birdsong | Mississippi. |
| B. D. Brabson | Tennessee. |
| W. H. Caffey | Alabama. |
| P. D. Cooper | Tennessee. |
| A. C. Drake | Kentucky. |
| A. A. Dyer | Texas. |
| Geo. A. Foster | Kentucky. |
| S. W. Foster | Alabama. |
| J. M. Hale | Tennessee. |
| E. T. Hardaway | Missouri. |
| Geo. A. Harper | Louisiana. |
| L. H. Henley | Alabama. |
| N. A. Neeley | Tennessee. |
| J. A. Parrish | Georgia. |
| Albert Robinson (honorary) | Michigan. |
| P. H. Wright | Mississippi. |

### Pennsylvania College of Dental Surgery.

The thirty-first annual commencement of the Pennsylvania College of Dental Surgery was held at the American Academy of Music, Philadelphia, on Saturday evening, February 26, 1887.

The degree of D.D.S. was conferred on the following graduates by S. W. Gross, M.D., President of the Board of Trustees:

| NAME. | RESIDENCE. |
|---|---|
| E. Herbert Adams | Canada. |
| Vittorio Adler, M. D. | Italy. |
| Holstein Bartilson | Pennsylvania. |
| J. M. Briggs | Georgia. |
| Reginald W. Bird | Kingston, Ja. |
| James M. Burke | New York. |
| Chas. W. Blind | Pennsylvania. |
| Carolina Bonin | Germany. |
| Luis Campuzano | Cuba. |
| John Robert Conway | Minnesota. |
| Woodfred E. Clayton | Canada. |
| G. Carrow Chance | Pennsylvania. |
| James A. Calhoon | Pennsylvania. |
| Lewis H. Chamberlin | New York. |
| Bellville M. Crary | Pennsylvania. |
| William P. Clarke | Pennsylvania. |
| R. Melvin Davenport | Massachusetts. |
| Elliott C. Douglass | Connecticut. |
| R. A. Dinsmore | Pennsylvania. |
| George B. Dodd | Wisconsin. |
| George C. Eighme | New York. |
| J. O. Ely | Pennsylvania. |
| P. Judson Eckel | New Jersey. |
| Howard T. Eachus | Pennsylvania. |
| Charles H. Fritts | Pennsylvania. |
| Paul F. Griselle | Ohio. |
| J. Howard Gaskill | Pennsylvania. |
| Alfred Gysi | Switzerland. |
| Frank S. Garrett | Pennsylvania. |
| J. W. Lambie | New York. |
| William C. Mason | Pennsylvania. |
| Wilhelm T. Muschan | Germany. |
| Frank H. McOmber | New York. |
| John H. McClure | West Va. |
| Jos. Elias Miller, M. D. | Pennsylvania. |
| William P. Noyse | Maine. |
| J. A. Neiman | Pennsylvania. |
| Milville S. Page | Connecticut. |
| Edson J. Quackenboss | Massachusetts. |
| Albert S. Rabenold | Pennsylvania. |
| Orville Rector | New York. |
| Lee Grant Richardson | New York. |
| A. Percy Roberts | Pennsylvania. |
| Joseph T. Rowand | California. |
| David Z. Sahler | Pennsylvania. |
| Peter Jos. H. Schwan | Germany. |
| George H. Seaver | New York. |
| William F. Shotts | Pennsylvania. |
| Casimir O. Shulthess | Switzerland. |
| Amos H. Sibley | Pennsylvania. |
| William B. Sickler | New Jersey. |
| George B. Smith | Ohio. |
| D. Arthur Smith | Pennsylvania. |
| Earnest A. Smith | N. B., Canada. |
| Edw. M. Stroud | Pennsylvania. |
| Robert H. Tecklenburg | Pennsylvania. |
| Carola Timmermann | Germany. |
| Charles M. Truesdel | Minnesota. |

| | |
|---|---|
| Lewis S. Goble | New Jersey. |
| Frank E. Hendrickson | New Jersey. |
| E. R. Hershey, M. D. | Pennsylvania. |
| Albert F. Hug | Illinois. |
| Charles S. Holden | Massachusetts. |
| Charles L. Hill | Pennsylvania. |
| Edward S. Jackson | Pennsylvania. |
| Adelheid Jacobi | Germany. |
| Charles G. Keeney | Pennsylvania. |
| Albert G. Koehler | Pennsylvania. |
| J. A. Kressly | Pennsylvania. |
| Joseph Vossen | Germany. |
| Walter R. Vrooman | Canada. |
| George W. Warren | New Jersey. |
| C. W. Wells, L. D. S. | Ontario, Can. |
| Walter Hart Williams | Pennsylvania. |
| Camille Wisner | France. |
| Perrie G. Wood | New York. |
| Frank L. Wright | Pennsylvania. |
| F. D. Yielding | Pennsylvania. |
| George B. Zell | Maryland. |

### Philadelphia Dental College.

The twent-fourth annual commencement of the Philadelphia Dental College was held at the Academy of Music, Philadelphia, on Friday evening, February 25, 1887.

The number of matriculates for the session was one hundred and ninety-four. The number of graduates was seventy-nine, as follows :

| NAME. | RESIDENCE. |
|---|---|
| Edson J. Abbey | Connecticut. |
| Eugene Bernhardt | Germany. |
| Clement E. Bill | Connecticut. |
| John A. Bolard | New Jersey. |
| Edward Bolard | Pennsylvania |
| H. E. Brady | Pennsylvania. |
| Asher S. Burton | New Jersey. |
| A. J. F. Buxbaum, M.D. | Ohio. |
| C. L. Card | New York. |
| J. de la Cerday Gomez | Spain. |
| F. N. Chamberlain | Ohio. |
| Mark O. Cooley | New York. |
| John C. Curry | New Jersey. |
| Harry P. Derr | Pennsylvania. |
| B. B. Detwiler | Virginia. |
| Ferdinand Dietzi | Switzerland. |
| Edgar L. Dow | Massachusetts. |
| William Dunn | Italy. |
| Hobart E. Duncan | Iowa. |
| L. V. W. Dupuis | Canada. |
| J. W. Erwin | Pennsylvania. |
| Albert P. Fellows | Kansas. |
| Frank E. Ferris | Iowa. |
| Samuel E. Frick | Pennsylvania. |
| Miles D. Glidden | Maine. |
| George W. Hall | New York. |
| Mark E. Harrison | Missouri. |
| John T. Hinch | Maine. |
| James A. Hodgins | Canada. |
| Herbert G. Hodgkins | Maine. |
| William D. Hughes | Pennsylvania. |
| Edgar L. Hurd | Maine. |
| Charles S. Inglis | New Jersey. |
| William T. Jackman | Ohio. |
| Charles W. Jarvis | Canada. |
| Frederick E. Judson | Minnesota. |
| George H. Judson | New York. |
| Albert C. Kellogg | Iowa. |
| Charles J. Kinkead | Delaware. |
| Sebastian Lacavelrie | Cuba. |
| Marshall H. Lamoree | New York. |
| Julian Landau | Germany. |
| Arthur W. S. Loewen | Pennsylvania. |
| Charles H. Lovejoy | Indiana. |
| Fred. Henry Lyder | Ohio. |
| J. Hyatt Lyke | New York. |
| Alex. Wm. Marshall | Australia. |
| Jacob Marquis | Pennsylvania. |
| Joseph McCreery, Jr | Pennsylvania. |
| Wilson Y. McGown | Maine. |
| Edward T. McNally | Massachusetts. |
| Hannah J. Mercer | Pennsylvania. |
| T. J. Morrison | Maine. |
| Karl R. Von Nauman | Austria. |
| Edward O'Neill | Pennsylvania. |
| P. T. O'Reilly | Massachusetts. |
| Charles L. Porter | Delaware. |
| James J Rafferty | Massachusetts. |
| A. Rogmans | Netherlands. |
| Joseph O. Rothwell | Ohio. |
| J. Antonio Serrano | U.S. Colombia. |
| J. Wesley Shaw | Massachusetts. |
| Orlando B. Shedd | New York. |
| George H. Smith | Pennsylvania. |
| John L. Stickel | Illinois. |
| P. F. Struppler | New York. |
| August Sulzberger | Switzerland. |
| Lot. D. Sutherland | New York. |
| Herbert S. Sutphen | New Jersey. |
| Ernest A. Tripp | Utah. |
| Charles L. Van Fossen | Kansas. |
| H. Bernhard Voigt | Germany. |
| Arthur E. Wales | Connecticut. |
| Miss Marion Ward | California. |
| George T. Ware | Canada. |
| Charles X. Weis | Connecticut. |
| George B. Williamson | Ohio. |
| T. Frank Windle | Pennsylvania. |
| William T. Wyckoff | New Jersey. |

### Annual Commencement of the University of Michigan.

The annual commencement of this Institution was held in the hall of the University on the 30th of June. During the preceding five days the semi-Centenniel of the University was celebrated, on which occasion thousands of the alumni were present and took part in the festivities. The Dental Department, though not celebrating its half century, yet the alumni took their part in the general festivities.

In the Dental College there have been in attendance ninety-six during the term. The following received the degree of Doctor of Dental Surgery:

| NAME. | NAME. |
|---|---|
| E. L. Avery. | Geo. H. Miner. |
| Ernest L. Avery. | Lewis H. McDonnald. |
| Frank C. Babcock. | Joseph L. Nordike. |
| Gilbert E. Corbin. | Edward E. Paxson. |
| Almon Dewhirst. | William A. Powers. |
| Elmer L. Drake. | William D. Saunders. |
| Fred. W. Gordon. | Frank L. Small. |
| David A. Harroun. | Eva C. Smith. |
| Harry D. Heller. | Clarence J. B. Stephens. |
| James B. Hoar. | James C. Stevens. |
| Almer M. Harrison. | Patrick J. Sullivan. |
| Fred. A. Kotts. | Charles H. Warboys. |
| Cyreno N. Leonard. | William A. Wright. |
| John T. Martin. | Edward L. Dillman. |

### Missouri Dental College.

The twenty-first annual commencement of the Missouri Dental College was held, in connection with that of the St. Louis Medical College, at Memorial Hall, St. Louis, Mo., on Wednesday evening, March 3, 1887.

The degree of D.D.S. was conferred on the following graduates by H. H. Mudd, M.D., Dean:

| NAME. | RESIDENCE. | NAME. | RESIDENCE. |
|---|---|---|---|
| Cline H. Bartlett | Missouri. | Albert E. Nichols | California. |
| Howard M. Combs | California. | Maurice W. Pearson | Missouri. |
| Edward G. Ellis | Missouri. | Sidney J. Smith | Missouri. |
| James A. Meng | Missouri. | | |

